# Practical applications of AI in body imaging

**DOI:** 10.1007/s00261-025-05088-3

**Published:** 2025-06-27

**Authors:** Benjamin M. Mervak, Jessica G. Fried, Julian Neshewat, Ashish P. Wasnik

**Affiliations:** University of Michigan-Ann Arbor, Ann Arbor, USA

**Keywords:** Artificial intelligence, Deep learning, Radiology, Workflow, Algorithms

## Abstract

Artificial intelligence (AI) algorithms and deep learning continue to change the landscape of radiology. New algorithms promise to enhance diagnostic accuracy, improve workflow efficiency, and automate repetitive tasks. This article provides a narrative review of the FDA-cleared AI algorithms which are commercially available in the United States as of late 2024 and targeted toward assessment of abdominopelvic organs and related diseases, evaluates potential advantages of using AI, and suggests future directions for the field.

**Supplementary Information:**

The online version contains supplementary material available at 10.1007/s00261-025-05088-3.

## Introduction/overview

Artificial intelligence (AI) has had a substantial impact in many areas of life and business in recent years, and healthcare is no exception. Machine learning (ML), a subclass of AI, uses techniques to create algorithms that allow predictions based on patterns in data [1]. Deep learning (DL), a subclass of ML, involves neural networks with multiple layers that can analyze numerous variables simultaneously to identify complex patterns in large datasets, such as imaging studies [1, 2]. In Radiology, these algorithms have shown the potential to enhance diagnostics, treatment planning, and operational efficiencies in medical settings [3]. In practice, these technologies have enabled the development of sophisticated tools for recognition of anatomical structures, segmentation of organs or parts, and detection of anomalies.

While it remains early in the process of development and implementation, some radiologic subspecialties have seen greater advances in AI applications than others. Abdominal radiology has lagged behind other subspecialties such as neuroradiology or cardiothoracic radiology [4, 5], although some algorithms for abdominal imagers have been developed and have received clearance from the U.S. Food and Drug Administration (FDA).

This manuscript provides a narrative review of the FDA-cleared AI algorithms which are commercially available in the United States as of late 2024 that are targeted toward assessment of abdominopelvic organs and related diseases. For the purposes of this paper, we use the term “AI” to mean ML or DL algorithms involved in the analysis of medical images for disease detection, lesion characterization, or organ segmentation. To narrow the scope of the paper, we assess only algorithms which are targeted to abdominopelvic organs in adults, focusing on applications in hepatobiliary, renal, bladder, prostate, and gastrointestinal imaging. Algorithms focused on tasks upstream of the radiologist (e.g., image acquisition), generation of reports (e.g., automatic impressions), or those focused on radiomics/feature extraction were not included in the scope. In addition to reviewing these algorithms, we illustrate how they can be useful in improving radiology workflows and discuss advantages of AI in this context. Lastly, we explore future directions for AI in abdominal radiology as this technology continues its rapid advancement.

## Methods/search strategy

To identify FDA-cleared AI algorithms relevant to abdominal imaging as of 9/11/2024, one abdominal imager with 8 years of post-fellowship experience (BMM) consulted the AI Central website hosted by the American College of Radiology Data Science Institute. This website catalogued 330 FDA-cleared or FDA-approved algorithms across all radiologic subspecialties [6]. To focus on algorithms pertinent to abdominal imagers, we selected the product category “abdominal imaging” which excluded 284 algorithms leaving 46 products of potential interest. All 46 results were further evaluated for their relevancy to abdominal imagers by evaluating the product details provided on the AI Central website. 22 additional algorithms were excluded as they met one or more of the following exclusion criteria: (1) algorithms focused on findings located outside of the abdomen (e.g., pulmonary nodules, neuroimaging algorithms, emboli at the lung bases, or vertebral body compression deformities), (2) algorithms focused on vascular findings (e.g., abdominal aortic aneurysm detection), (3) algorithms intended to run at the time of image acquisition (e.g., gauging image quality or modifying magnetic resonance imaging sequences as they are acquired), (4) algorithms for pediatric or fetal imaging, (5) algorithms with outputs targeted at non-radiologist physicians (e.g., creation of 3D-printed models for surgeons). Any uncertainties on whether an algorithm should be included or excluded was adjudicated by three abdominal imagers (BMM, JGF, and APW). A total of 24 algorithms met inclusion criteria (Table 1).

**Table 1:** FDA-cleared AI algorithms relevant to abdominal imaging that Met inclusion criteria as of 9/11/2024

Algorithm name	Manufacturer	Organ system	Modality	Category	Date cleared	ACR transparent AI certified?	Interface and output(s)
Advantis platform	Advantis Medical Imaging	Prostate	MR	MIMPS	3/1/2023	–	Viewer, automated/structured reporting
AI metrics	AI Metrics	Multiple	CT, MR	MIMPS	12/22/2020	–	Viewer, automated/structured reporting
AI-Rad Companion Prostate MR	Siemens Healthineers	Prostate	MR	MIMPS	7/30/2020	Yes	Viewer, prostate contours, structured reporting
Briefcase Intra-abdominal Free Gas Triage	Aidoc Medical	Peritoneum	CT	CADt	6/19/2020	Yes	Viewer, pop up notification
Change Healthcare Anatomical AI	Change Healthcare	Multiple	CT, MR	MIMPS	7/20/2021	–	JSON file with anatomic regions
CoverScan	Perspectum Diagnostics Ltd.	Multiple	MR	MIMPS	3/3/2023	–	Quantitative report (Liver fat and cT1, pancreas PDFF and T1, spleen length, renal T1 and length)
DeepLook PRECISE	DeepLook	Multiple	CT, MR, US, XR	MIMPS	4/9/2021	–	Viewer, ROI segmentation and dimensions
FerriSmart Analysis System	Resonance Health Analysis Service	Liver	MR	MIMPS	11/30/2018	–	Quantitative report (LIC)
GIQuant	Motilent	Small bowel	MR	MIMPS	11/8/2021	–	Quantitative report (bowel motility)
HealthFLD	Nanox	Liver	CT	MIMPS	2/8/2024	–	Viewer, liver attenuation value (HU), notifications
HepaFatSmart	Resonance Health Analysis Service	Liver	MR	MIMPS	6/20/2023	–	Quantitative report (VLFF, PDFF, steatosis grade)
Hepatic VCAR	GE Healthcare	Liver	CT	MIMPS	3/20/2020	–	Viewer, segmentation (liver, lesion, vasculature)
Hepatica	Perspectum Diagnostics Ltd.	Liver	MR	MIMPS	1/12/2021	Yes	Quantitative report (liver volumetry, cT1, and PDFF)
Liver Suite	GE Healthcare	Liver	CT	MIMPS	5/2/2022	–	Viewer, guided workflow, structured reporting
Liver Surface Nodularity	Imaging Biometrics	Liver	CT	MIMPS	10/29/2020	–	Viewer, liver nodularity score
LiverMultiScan	Perspectum Diagnostics Ltd.	Liver	MR	MIMPS	9/6/2022	Yes	Quantitative report (cT1, PDFF, LIC)
LiverSmart	Resonance Health Analysis Service	Liver	MR	MIMPS	12/29/2021	–	Quantitative report (LIC, VLFF, PDFF, steatosis grade)
Medihub Prostate	JLK Inc.	Prostate	MR	MIMPS	6/21/2024	–	Viewer, prostate contours, structured reporting
MRCP+	Perspectum Diagnostics Ltd.	Biliary	MR	MIMPS	3/13/2024	Yes	Quantitative report (biliary volume, ducts, strictures, dilations, and model)
Prostat ID	Botimageai	Prostate	MR	CADe/x	7/8/2022	–	Viewer, quantitative report (prostate volume, suspected lesions)
PROView DL	GE Healthcare	Prostate	MR	MIMPS	11/17/2020	–	Viewer, quantitative report (prostate volume, PSA density, lesion ROI, lesion mapping)
qp-Prostate	Quibim	Prostate	MR	MIMPS	2/4/2021	–	Viewer, quantitative report (prostate volume, lesion map)
Quantib Prostate	Quantib BV	Prostate	MR	MIMPS	4/17/2023	–	Viewer, quantitative report (prostate volume, PSA density, lesion map)
StoneChecker	Imaging Biometrics	Kidney	CT	MIMPS	9/26/2019	–	Viewer, quantitative report (ROI density/texture values and measurements)

Information provided by the AI central website hosted by the American college of radiology data science institute

*MR* magnetic resonance imaging, *CT* computed tomography, *US* ultrasound, *XR* radiography, *MIMPS* medical image management and processing systems, *CADt* computer-aided triage and notification, *cT1 *corrected T1, *PDFF* proton density fat fraction, *VLFF* volumetric liver fat fraction, *LIC* liver iron concentration, *HU* Hounsfield units, *ROI* region of interest

The ACR AI Central website and 501(k) premarket notification documents were used to gather additional information on algorithms including the organ system of focus, modalities included, category of algorithm, and date of FDA clearance. Several algorithms were found to participate in a program created by the ACR called “Transparent AI” which requires the voluntary submission of data elements by manufacturers, including data on model identification, characteristics, indications for use, performance, training details, and limitations. Finally, the manufacturer’s website, any available Instructions for Use (IFU) documents, and any online demonstration images or videos were consulted for each algorithm to determine the type of interface and output of data (Table 1).

## Abdominal imaging focused algorithms

### Hepatobiliary

The increasing global prevalence of liver diseases highlights the critical need for precise diagnosis, characterization, and staging of hepatobiliary disorders. AI algorithms have demonstrated significant potential in identifying patterns, automating diagnostic processes, and enhancing clinical decision-making in the management of hepatobiliary conditions. These models have proven effective in detecting and staging chronic liver diseases including metabolic dysfunction-associated steatotic liver disease (MASLD), as well as in quantifying liver fat and iron content, or diagnosing cirrhosis and hepatocellular carcinoma. Some of the FDA approved AI models for clinical applications are discussed here.

Multiparametric MRI is now an acceptable noninvasive method for liver fat quantification and fibrosis using proton density fat fraction (PDFF) maps, T2*, and iron-corrected T1 (cT1, necessary to correct the confounding effect of excess iron on T1) [7–9]. AI algorithm-based software applications used with a supported MR scanner can automatically calculate cT1 from T1 and T2* maps (Liver MultiScan, Perspectum Diagnostics, Oxford, UK) [8–10]. While some vendors may offer proprietary solutions providing fat or fibrosis quantification, third party AI applications may be advantageous to healthcare systems employing multiple brands of MR scanners as the algorithm is vendor neutral and makes use of existing modality software and hardware to run a sequence roughly 5 min in length [11]. Banerjee et al. demonstrated the high diagnostic accuracy of this non-invasive approach in detecting hepatic fibrosis compared to the gold standard, histology [8]. Bachtiar et al. showed a repeatability coefficient of 0.8% (i.e., maximum expected difference of 0.8% likely to occur between repeated measurements) and reproducibility coefficient of 0.75% (i.e., maximum expected difference of 0.75% likely to occur between measurements made by different observers or in different conditions) for hepatic fat based on PDFF across 1.5 and 3 T field strengths and various manufacturers [10]. It has also been shown to be effective in diagnosing MASLD and identifying patients with metabolic-dysfunctional associated steatohepatitis (MASH) and cirrhosis [12, 13]. Additionally, cT1 can serve as a biomarker to inform the management of chronic liver diseases including autoimmune hepatitis and viral hepatitis [14, 15]. In patients with chronic liver disease, the risk of morbidity and mortality increases significantly as cirrhosis progresses [16]. Although liver biopsy is the gold standard for diagnosing cirrhosis, given the invasive nature it carries the risk of complications [17]. As a result, there is an increasing interest in developing non-invasive methods for accurately diagnosing cirrhosis.

Various imaging features assessed on ultrasound (US), computed tomography (CT), and magnetic resonance imaging (MRI), such as liver volume ratio, splenic volume, and liver surface nodularity have been studied for their correlation with cirrhosis. Among these, severe liver surface nodularity is particularly strongly associated with cirrhosis [18–20], and surface nodularity scoring on CT has shown notable diagnostic accuracy in detecting advanced fibrosis and cirrhosis [21, 22]. Liver Surface Nodularity (LSN), (Imaging Biometrics, LLC, Elm Grove, WI) is an FDA-cleared semi-automated CT software tool that aids in analyzing and reporting liver surface nodularity for the evaluation of chronic liver disease. The software works with Digital Imaging and Communications in Medicine (DICOM) format CT images in a viewer, where the user delineates 5–10 linear regions of interest (ROIs) along the anterior edge of the liver. The software then automatically detects the liver surface on the selected slice and adjacent contiguous slices, generating a final liver surface nodularity (LSN) score by calculating the arithmetic mean of the measurements from each slice [18, 23]. Lubner et al. demonstrated that LSN assessment on CT can accurately and noninvasively quantify liver surface nodularity, correlating it with intermediate fibrosis stages in patients with hepatitis C virus (HCV) [24]. In a multi-institutional study of 161 patients with chronic liver disease from HCV, the AUC of the LSN score alone was 0.87 for detecting advanced fibrosis (≥ F3) and 0.89 for detecting cirrhosis (F4); the LSN score was the strongest predictor of baseline liver decompensation in multivariate analysis [22].

ML models have also been shown to automatically analyze multi-slice, spin-echo MRI datasets of the abdomen and calculate the signal decay rate (R2), a parameter used to assess liver iron content. FerriSmart Analysis System (Resonance Health, Burswood, Western Australia) is one such software that can be deployed on a cloud-based platform or locally, and applies a predefined calibration curve to the data, providing a quantitative measurement of liver iron concentrations in vivo [25].

Additionally, there are FDA approved post-processing applications like Liver Suite (GE HealthCare, Chicago, IL**)** which can assist in evaluating liver lesions on CT in patients with known or suspected hepatocellular carcinoma (HCC) by offering optimized series display and a guided workflow to assess key clinical features for user-defined observations, facilitating the computation of a Liver Imaging Reporting & Data System (LI-RADS) score [26].

Magnetic Resonance Cholangiopancreatography (MRCP) is a non-invasive imaging technique used for evaluating the biliary tract in diagnosing and monitoring primary sclerosing cholangitis, both with and without associated biliary cirrhosis. MRCP has higher sensitivity in identifying biliary strictures and dilation; however, accurate assessment is crucial for identifying disease progression or the development of neoplasms. Due to high inter-reader variability, an objective and standardized approach to interpretation is essential [27]. An AI-driven semiautomated tool, MRCP+ (Perspectum Diagnostics, Oxford, UK), has been shown to extract quantitative metrics, allowing for the reconstruction of a 3D model of the biliary tree. The software calculates various measurements, including bile duct diameter, the severity of biliary dilation, and the percentage of biliary ductal stenosis from non-contrast MRCP images [28, 29]. These metrics have been shown to correlate with biochemical, elastographic, and radiological prognostic scores, and they are predictive of adverse outcome-free survival in primary sclerosing cholangitis [30].

### Renal

Renal stones are commonplace in abdominal imaging as nearly 10% of people in the United States are affected by renal stone disease [31]. Accurate quantification and characterization of kidney stones are crucial for determining appropriate treatment strategies, which can range from conservative management to surgical intervention. Traditional methods of evaluating kidney stones via computed tomography (CT) scans present challenges, including variability in interpretation among radiologists and the labor-intensive nature of manually analyzing multiple parameters. One algorithm (StoneChecker, Imaging Biometrics, LLC, Elm Grove, WI) can help reduce the burden on radiologists by calculating stone volume, mean Hounsfield unit (HU) density, skin-to-stone distance, and various texture values (mean, mean of positive pixels, standard deviation, skewness, kurtosis, and entropy), thereby helping to standardize interpretation and guide treatment decisions. The product analyzes DICOM CT images and generates output metrics like stone volume and HU density to permit assessment of the stone’s composition and likelihood of successful conservative management [32]. Texture analysis provides additional insights into the stone’s internal composition, which can aid in predicting the success of lithotripsy, or predict the number of shocks needed to fragment the stone [33]. In one small clinical study of 45 patients, the algorithm was found to reliably provide texture analysis to help differentiate between softer uric acid stones and harder non-uric acid stones with single-feature areas under the curve ranging from 0.898 to 0.960 [34].

### Prostate

MRI is an established imaging modality for the noninvasive detection of clinically significant prostate cancer. To our knowledge, as of late 2024 there were no AI algorithms in the United States which were FDA-cleared for lesion detection, although some are available in Europe and several vendors report pursuing clearance in the US. Several ML models have shown promising results in assisting radiologists with the assignment of Prostate Imaging Reporting & Data System (PI-RADS) categories. Products can also help provide semi-automated segmentation of observations to permit MR-US fusion biopsy. Some of the FDA approved AI based software utilize U-Net-like convolutional neural network (CNN) models, for example QP-Prostate (Quibim, Valencia, Spain), deep neural networks, for example AI-Rad Companion Prostate MR (Siemens Healthineers, Erlangen, Germany), or a semi-automated approach like Quantib Prostate, *(*Quantib B.V., Rotterdam, The Netherlands) for guiding risk stratification using PI-RADS v2.1 [35–38]. Software packages can also automatically measure the prostate gland volume, PSA density, and assist with structured reporting. Studies have shown that the software enhances diagnostic accuracy in detecting suspicious prostate lesions (with PI-RADS 4 detection improving by 4.4%, increasing the area under the curve from 0.84 to 0.88), reduces inter-reader variability (inter-reader concordance improved by 63%), and decreases reading time (median reading time reduced by 21%, from 103 s to 81 s) [37, 39]. In a study assessing the clinical utility of such software, comparisons between analyses conducted by inexperienced and experienced radiologists revealed a sensitivity of 92.3% for patients with positive MRI and positive biopsy results [38].

### Gastrointestinal

MR Enterography (MRE) is an established imaging technique for diagnosing and monitoring small-bowel Crohn’s disease. Research has demonstrated a negative correlation between terminal ileum motility and disease activity, meaning bowel motility declines as intestinal inflammation increases [40, 41]. As a result, objective and quantifiable measures of intestinal motility can serve as a biomarker for treatment response in Crohn’s disease. GIQuant (Motilent Ltd., London, UK) is an AI-driven postprocessing software that uses dynamic cine images from MRE to assess small bowel motility. The algorithm creates a motility map and anatomical reference image from each dynamic sequence, which are then used to calculate a motility score [41].

### Workflow alteration

Some algorithms are designed to modify and assist a radiologist’s workflow with the goal of increasing efficiency and/or improving patient care. One common area of focus for algorithms in this category is oncologic imaging and follow-up, representing a large portion of the routine work for abdominal imagers. These algorithms can also provide support for clinical trials in which specific target lesions are systematically tracked over time.

One feature of several workflow-alteration algorithms includes automated measurement and segmentation of lesions. Utilizing an algorithm may allow for more streamlined point-and-click functionality to measure lesions in 2 or 3 dimensions or enable volumetric segmentation of lesions. This further may allow for easier tracking of lesion size over time and the generation of tables or graphs to be embedded in radiologic reports. Examples of currently FDA-cleared algorithms in this space include AI Metrics (AI Metrics, Birmingham, AL), and DL Precise (Deeplook Medical, New Haven, CT).

AI algorithms can also be directly embedded in commonly used Radiology IT software systems, for example the picture archiving and communications system (PACS), and can be used to supplement workflows before or while a radiologist views a study. For example, some vendors have introduced AI-enhanced methods for identifying relevant prior studies and ensuring that they are displayed or available for review during the interpretation of an examination. One example of an FDA-cleared algorithm providing this functionality is Anatomical AI (Change Healthcare, Nashville, TN).

## Opportunities and future directions of AI applications in abdominal imaging

### Opportunities

Artificial intelligence algorithms are marketed as offering several advantages over those practicing without using AI. Although few studies have empirically measured the size of these advantages, particularly for abdominal imagers, radiologists expect AI to offer benefits like improved efficiency (e.g., from automated hanging protocols, segmentation of organs, segmentation of lesions, or in report generation) or improved diagnostic accuracy (e.g., lesion detection, lesion characterization, or quantitative assessment) [5, 42–45].

A reduction in repetitive tasks would increase efficiency and be an improvement in the quality of life for many radiologists. Narrow (focused) AI algorithms excel at tasks like this, and we have listed several of the commercially available algorithms that can support abdominal imagers by performing automated segmentation of organs (e.g., liver, biliary tree, or prostate), or save time by allowing point-and-click orthogonal or multiplanar analysis of lesions [46].

As detailed above, other algorithms aim to increase the diagnostic accuracy of abdominal imagers or improve quantitative assessment. Algorithms can help add objectivity in measuring liver fat, iron, fibrosis, or nodularity instead of relying on more subjective anatomical changes or differences between sequences or can provide decision support when assigning PI-RADS scores to prostate lesions.

### Future directions

While progress has been made in the field imaging artificial intelligence as it pertains to abdominal imaging, this remains an area in which we will likely see great future growth. Particularly in comparison to other radiologic subspecialties like neuroradiology or cardiothoracic radiology, bench to bedside product realization has been relatively slow and meager for abdominal imaging applications [47]. Beyond continued development of highly reliable algorithms in domains discussed in this manuscript as well as in other clinical areas with currently unmet needs, future directions for artificial intelligence in abdominal imaging include radiomics, clinical decision support, and workflow/patient throughput.

Radiomics involves the extraction of quantitative data from medical images that is not readily apparent to human perception to improve diagnostic and prognostic accuracy of imaging interpretation. Despite promising research demonstrating the potential for radiomics to improve diagnostic differentiation, treatment selection, and prognostic outcomes in hepatocellular carcinoma, colorectal cancer, renal tumors, and pancreatic ductal adenocarcinoma, these techniques have not been readily translated into clinical practice in body imaging [48]. The multi-step effort-intensive process involves image segmentation, texture feature extraction, data analysis, prediction model construction, and validation. Continued advancements in efficiency and fidelity of image segmentation will address a critical current barrier to integrating radiomics into clinical practice.

An exciting emerging focus of artificial intelligence development is clinical decision support. Clinical decision support artificial intelligence models are designed to integrate prior imaging findings, laboratory results, and clinical information to assist in providing comprehensive patient assessments. These models have shown promise in other medical fields and may have the potential to streamline the radiologist’s workflow and enhance image interpretation [49]. Eliminating the potential time sink spent on contextualizing each imaging exam at the time of interpretation could improve radiologist efficiency and condensed relevant medical information may improve diagnostic accuracy, particularly in abdominal imaging where patient history, prior treatments, and laboratory studies can play a substantial role in arriving at the correct diagnosis and/or prioritizing differentials.

Additional emerging areas of applications of artificial intelligence in imaging that could benefit abdominal imaging are in the domain of improving workflows and patient throughput. Reducing magnetic resonance scan times by employing deep learning algorithms to reconstruct high-quality images from lower quality data may improve patient throughput. Artificial intelligence algorithms to improve scheduling efficiency may also improve patient throughput through all modalities relevant to abdominal imaging. Algorithms could conceivably be developed to maximize asset/resource utilization and minimize asset downtime [50]. Although currently paused, the embattled Protecting Access to Medicare Act (PAMA) appropriate use program designed to reduce inappropriate advanced imaging ordering could witness a renaissance ushered by advancements in artificial intelligence. Later iterations of some clinical decision support mechanisms (e.g., CareSelect [Change Healthcare, Nashville, TN]) employed natural language processing and rudimentary artificial intelligence to allow users to input diagnostic queries and return suggested appropriate imaging [51]. The appropriate use criteria program is currently undergoing re-evaluation with some indication that implementation efforts will eventually recommence. Sophisticated AI algorithms could potential succeed where past efforts have failed to reduce inappropriate advanced diagnostic imaging, which would have broad implications on abdominal imaging which sees a high volume of CT and MRI. While not specific to abdominal imaging, advancement in artificial intelligence applications in these areas could improve practice generally.

Future algorithms in abdominal imaging will likely operate across organ systems, grow computer-aided detection/diagnosis applications, and expand into additional organ systems. Most abdominal imaging AI algorithms to date have focused on narrow task functions—addressing a single specific task, focused on a single organ or pathologic entity, commonly on a single imaging modality. Ideally, future research should focus on comprehensive AI solutions, aiming to simulate human level-intelligence. Comprehensive AI solutions may be more applicable to abdominal imaging than traditional narrow-task AI, given the multi-modality nature, complex underlying anatomy, and nuanced interconnected pathologies seen in abdominal imaging.

## Conclusion

AI has begun to change the practice of radiology, including in abdominal imaging. As AI is increasingly implemented, radiologists leveraging AI tools may find benefits in improving diagnostic accuracy, streamlining workflows, and automating repetitive tasks. While still in its infancy with abdominal imaging, this review highlights 28 currently available (FDA-cleared) AI applications for abdominal imagers, discussing their capabilities and the potential benefits they offer in clinical practice. It is important to note that gaps still exist between the theoretical benefits of AI tools and the real-world experience with what is currently commercially available [4, 52]. Specific to lesion detection and characterization in abdominal radiology, while research has shown potential in many organ systems, few applications have been incorporated into FDA-cleared solutions. AI offerings in abdominal imaging lag behind several other radiologic subspecialties [53], although many new algorithms become FDA cleared each year and we anticipate a future increase in the number of options available to abdominal imagers.

While several benefits exist, the implementation of AI in abdominal imaging also involves challenges and risks including potential biases in training data, model drift, inherent ‘black box’ nature of some algorithms, and ethical considerations regarding patient data privacy and liability. To maximize the benefits of AI while mitigating these risks, local validation of algorithms, continuous monitoring, and governance are needed.

Future advancements may focus on increasing translation from “bench to bedside,” and growing the number of FDA-cleared AI algorithms for abdominal imagers, developing comprehensive clinical decision support models, and integrating multifactorial patient data to provide holistic assessments.

## Electronic supplementary material

Below is the link to the electronic supplementary material.

Supplementary Material 1

## Data Availability

No datasets were generated or analysed during the current study.
