# Supplementary material for: Practical applications of AI in body imaging
Source: Abdom Radiol (NY). 2025 Jun 27;51(1):458–66. doi: 10.1007/s00261-025-05088-3 (PMC12830429; doi:10.1007/s00261-025-05088-3)
Supplement: Supplementary file 1 — Supplementary Material 1 [file 261_2025_5088_MOESM1_ESM.docx]

**Table 1.** FDA-cleared AI algorithms relevant to abdominal imaging that met inclusion criteria as of 9/11/2024. Information provided by the AI Central website hosted by the American College of Radiology Data Science Institute.

MR = Magnetic resonance imaging; CT = computed tomography; US = ultrasound; XR = radiography; MIMPS = Medical Image Management and Processing Systems; CADt = computer-aided triage and notification; cT1 = corrected T1; PDFF = proton density fat fraction; VLFF = volumetric liver fat fraction; LIC = liver iron concentration; HU = Hounsfield units; ROI = region of interest.

| **Algorithm Name** | **Manufacturer** | **Organ System** | **Modality** | **Category** | **Date Cleared** | **ACR Transparent AI Certified?** | **Interface and Output(s)** |
| --- | --- | --- | --- | --- | --- | --- | --- |
| Advantis Platform | Advantis Medical Imaging | Prostate | MR | MIMPS | 3/1/2023 | - | Viewer, automated/structured reporting |
| AI Metrics | AI Metrics | Multiple | CT, MR | MIMPS | 12/22/2020 | - | Viewer, automated/structured reporting |
| AI-Rad Companion Prostate MR | Siemens Healthineers | Prostate | MR | MIMPS | 7/30/2020 | Yes | Viewer, prostate contours, structured reporting |
| Briefcase Intra-abdominal Free Gas Triage | Aidoc Medical | Peritoneum | CT | CADt | 6/19/2020 | Yes | Viewer, pop up notification |
| Change Healthcare Anatomical AI | Change Healthcare | Multiple | CT, MR | MIMPS | 7/20/2021 | - | JSON file with anatomic regions |
| CoverScan | Perspectum Diagnostics Ltd. | Multiple | MR | MIMPS | 3/3/2023 | - | Quantitative report (Liver fat and cT1, pancreas PDFF and T1, spleen length, renal T1 and length) |
| DeepLook PRECISE | DeepLook | Multiple | CT, MR, US, XR | MIMPS | 4/9/2021 | - | Viewer, ROI segmentation and dimensions |
| FerriSmart Analysis System | Resonance Health Analysis Service | Liver | MR | MIMPS | 11/30/2018 | - | Quantitative report (LIC) |
| GIQuant | Motilent | Small bowel | MR | MIMPS | 11/8/2021 | - | Quantitative report (bowel motility) |
| HealthFLD | Nanox | Liver | CT | MIMPS | 2/8/2024 | - | Viewer, liver attenuation value (HU), notifications |
| HepaFatSmart | Resonance Health Analysis Service | Liver | MR | MIMPS | 6/20/2023 | - | Quantitative report (VLFF, PDFF, steatosis grade) |
| Hepatic VCAR | GE Healthcare | Liver | CT | MIMPS | 3/20/2020 | - | Viewer, segmentation (liver, lesion, vasculature) |
| Hepatica | Perspectum Diagnostics Ltd. | Liver | MR | MIMPS | 1/12/2021 | Yes | Quantitative report (liver volumetry, cT1, and PDFF) |
| Liver Suite | GE Healthcare | Liver | CT | MIMPS | 5/2/2022 | - | Viewer, guided workflow, structured reporting |
| Liver Surface Nodularity | Imaging Biometrics | Liver | CT | MIMPS | 10/29/2020 | - | Viewer, liver nodularity score |
| LiverMultiScan | Perspectum Diagnostics Ltd. | Liver | MR | MIMPS | 9/6/2022 | Yes | Quantitative report (cT1, PDFF, LIC) |
| LiverSmart | Resonance Health Analysis Service | Liver | MR | MIMPS | 12/29/2021 | - | Quantitative report (LIC, VLFF, PDFF, steatosis grade) |
| Medihub Prostate | JLK Inc. | Prostate | MR | MIMPS | 6/21/2024 | - | Viewer, prostate contours, structured reporting |
| MRCP+ | Perspectum Diagnostics Ltd. | Biliary | MR | MIMPS | 3/13/2024 | Yes | Quantitative report (biliary volume, ducts, strictures, dilations, and model) |
| Prostat ID | Botimageai | Prostate | MR | CADe/x | 7/8/2022 | - | Viewer, quantitative report (prostate volume, suspected lesions) |
| PROView DL | GE Healthcare | Prostate | MR | MIMPS | 11/17/2020 | - | Viewer, quantitative report (prostate volume, PSA density, lesion ROI, lesion mapping) |
| qp-Prostate | Quibim | Prostate | MR | MIMPS | 2/4/2021 | - | Viewer, quantitative report (prostate volume, lesion map) |
| Quantib Prostate | Quantib BV | Prostate | MR | MIMPS | 4/17/2023 | - | Viewer, quantitative report (prostate volume, PSA density, lesion map) |
| StoneChecker | Imaging Biometrics | Kidney | CT | MIMPS | 9/26/2019 | - | Viewer, quantitative report (ROI density/texture values and measurements) |
